# Numerical modeling of MR angiography for validation of image-driven quantitative diagnosis of intracranial aneurysm and carotid stenosis

**DOI:** 10.1186/2197-7364-1-S1-A63

**Published:** 2014-07-29

**Authors:** Artur Klepaczko, Piotr Szczypiński, Michał Strzelecki, Andrzej Materka

**Affiliations:** Institute of Electronics, Lodz University of Technology, ul. Wolczanska 211/215, 90-924 Lodz, Poland

This study aims to establish a numerical framework for validation of methods of quantitative analysis of non-invasive MR angiography imaging protocols such as Time-of-Flight (ToF) and Phase Contrast Angiography (PCA). In consequence, it is expected to reliably and objectively verify blood flow and volume measurements derived from image data.

The blood flow rate and volume are important predictors in diagnosing of cerebral aneurysms and carotid artery stenosis. Within the MRA setting, blood velocity can be directly measured from PCA images, while volume of an organ vasculature can be inferred from ToF data by applying vessel segmentation algorithms. Credibility of the methods is verified by comparing their results against reference models designed using MRA imaging simulator.

The fluid dynamics is simulated in the Comsol Multiphysics software based on Navier-Stokes equation and assuming the laminar regime. In the conducted experiments the blood motion was forced by setting a pressure difference between inlets and outlets. This difference was varied across a series of trials. Also, the size of aneurysm dome and narrowing was varied to observe influence of lesion geometry on velocity profile.

The MRA simulator implements a 3D Spoiled Gradient Echo sequence with optional flow compensation mechanism. Apart from information on velocity, blood spins are assigned magnetization vectors, whose evolution is determined based on the Bloch equation. Contributions from all flowing spins through are integrated in k-space and then transformed using the inverse FFT.

The synthesized PCA images were processed to extract local blood velocity. The results reveal agreement between PCA image-derived velocity values and CFD-simulated data in regions of unperturbed flow. In aneurysm and stenosis regions, there appear significant discrepancies between measurements and reference model. Based on ToF images, vessels and aneurysm dome diameters were determined. Again, in the lesion areas underestimation of regional blood volume is observed.

